# Single‐Ion Lithium Conducting Polymers with High Ionic Conductivity Based on Borate Pendant Groups

**DOI:** 10.1002/anie.202114024

**Published:** 2021-12-29

**Authors:** Gregorio Guzmán‐González, Soline Vauthier, Marta Alvarez‐Tirado, Stéphane Cotte, Laurent Castro, Aurélie Guéguen, Nerea Casado, David Mecerreyes

**Affiliations:** ^1^ POLYMAT University of the Basque Country UPV/EHU Avenida Tolosa 72 20018 Donostia-San Sebastián Spain; ^2^ Advanced Material Research Battery & Fuel Cell Toyota Motor Europe Research & Development 1 1930 Zaventem Belgium; ^3^ IKERBASQUE, Basque Foundation for Science 48011 Bilbao Spain

**Keywords:** Boron-Based Polymers, Lithium Batteries, Polymer Electrolytes, Polymerizable Boron-Lithium Salts

## Abstract

A family of single‐ion lithium conducting polymer electrolytes based on highly delocalized borate groups is reported. The effect of the nature of the substituents on the boron atom on the ionic conductivity of the resultant methacrylic polymers was analyzed. To the best of our knowledge the lithium borate polymers endowed with flexible and electron‐withdrawing substituents presents the highest ionic conductivity reported for a lithium single‐ion conducting homopolymer (1.65×10^−4^ S cm^−1^ at 60 °C). This together with its high lithium transference number *t* ${}_{\mathrm{Li}{}^{+}}$ =0.93 and electrochemical stability window of 4.2 V vs Li^0^/Li^+^ show promise for application in lithium batteries. To illustrate this, a lithium borate monomer was integrated into a single‐ion gel polymer electrolyte which showed good performance on lithium symmetrical cells (<0.85 V at ±0.2 mA cm^−2^ for 175 h).

## Graphical abstract

A new family of lithium‐conducting methacrylic polymer electrolytes based on highly delocalized borate groups is discussed. The optimized borate homopolymer shows the highest ionic conductivity reported for a single‐ion conducting polymer electrolyte for lithium metal batteries.

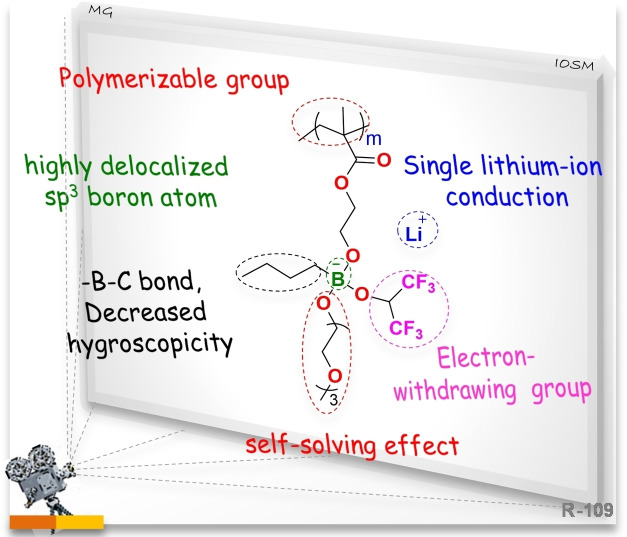

Single lithium‐ion conducting polymer electrolytes (SLICPEs) have been proposed as one promising solid electrolyte solution to overcome premature failures in solid‐state lithium metal batteries.[^1^, ^2^] Single‐ion conductors show limited formation of ionic concentration gradients in the electrolyte, which avoids dendritic growth on the lithium anode surface.[^3^, ^4^] Unlike classical solid polymeric electrolytes (SPEs) based on lithium salts dissolved in polymeric matrices such as PEO,^[5]^ in single‐ion conductors the anion moiety is chemically attached to the polymeric backbone and only the lithium counter‐cations are fully mobile. As a consequence, single‐ion conductors typically show lithium transfer numbers (LTNs) (close to unity) higher than those observed in dual‐ion conduction SPEs (between 0.2 and 0.5).^[6]^ However, they typically show low ionic conductivities vs. typical dual ion polymer electrolytes due to reduced concentration of free ionic species and the limited mobility of the lithium cation vs. mobile anions.

To date, several polymer chemistries have been proposed and explored for the preparation of single‐ion polymer electrolytes. In most cases, the anionic functional groups attached to the polymer backbones are carboxylates, sulfonates,^[7]^ sulfonamides or tetrahedral borates.[^8^, ^9^] It has been observed that anions with high charge delocalization are preferred to obtain high ionic conductivity since the ionic association with the lithium ions is weakened and the mobility of the lithium cation is improved.^[10]^ The highest ionic conductive polymers are obtained with anions like sulfonamides or tetrahedral borates which have reported ionic conductivity values less 1×10^−6^ S cm^−1^, e.g. Poly(LiMTFSI),[^11^, ^12^] and Poly(STFSI)[^13^, ^14^] with 1×10^−12^, and 7.6×10^−6^ S cm^−1^ at 25 °C, respectively. These values of ionic conductivity in homopolymers remain low for proper battery operation. For this reason, ionic transport and the ionic conductivity values are increased by formulation of the SLIPCE with plasticizers,[^2^, ^15^] blending with flexible polymeric matrices such as PEO^[16]^ or block copolymerization.[^17^, ^18^, ^19^] The main objective of this work is to report the synthesis of an innovative family of anionic monomers based on the highly delocalized asymmetric borate group and their homopolymers, which show very high ionic conductivity for a single‐ion polymer electrolyte.

In Figure 1, a general scheme for the methacrylic borate lithium salts and a picture of the polymer electrolyte membrane is presented. The chemical structures of these polymerizable boron–lithium salts was designed to integrate an ethoxy methacrylate group, a butyl group via a (B−C−) linkage, and two oxy (B‐OR) substituents. These serve as flexible polymerizable arm, agent to stabilization‐decreasing hygroscopicity,[^20^, ^21^] and modulation of the electron‐withdrawing capacity of the borate groups,^[22]^ respectively. The methacrylic borate SLICPEs synthesis involved three steps. In the first step, 2‐hydroxyethyl methacrylate is covalently bonded to the boron atom by a −C−O−B− bond. In the second stage, nBuLi is added to the boron atom to give rise to the formation of the boron–lithium salts LBB(OR)_2_, containing a covalent bond (−C−B−). Finally, the different monomers were polymerized by a conventional free radical polymerization method. Figure 2 shows the synthesis route and chemical structure for the eight different borate methacrylic homopolymers investigated in this work.

**Figure 1:**
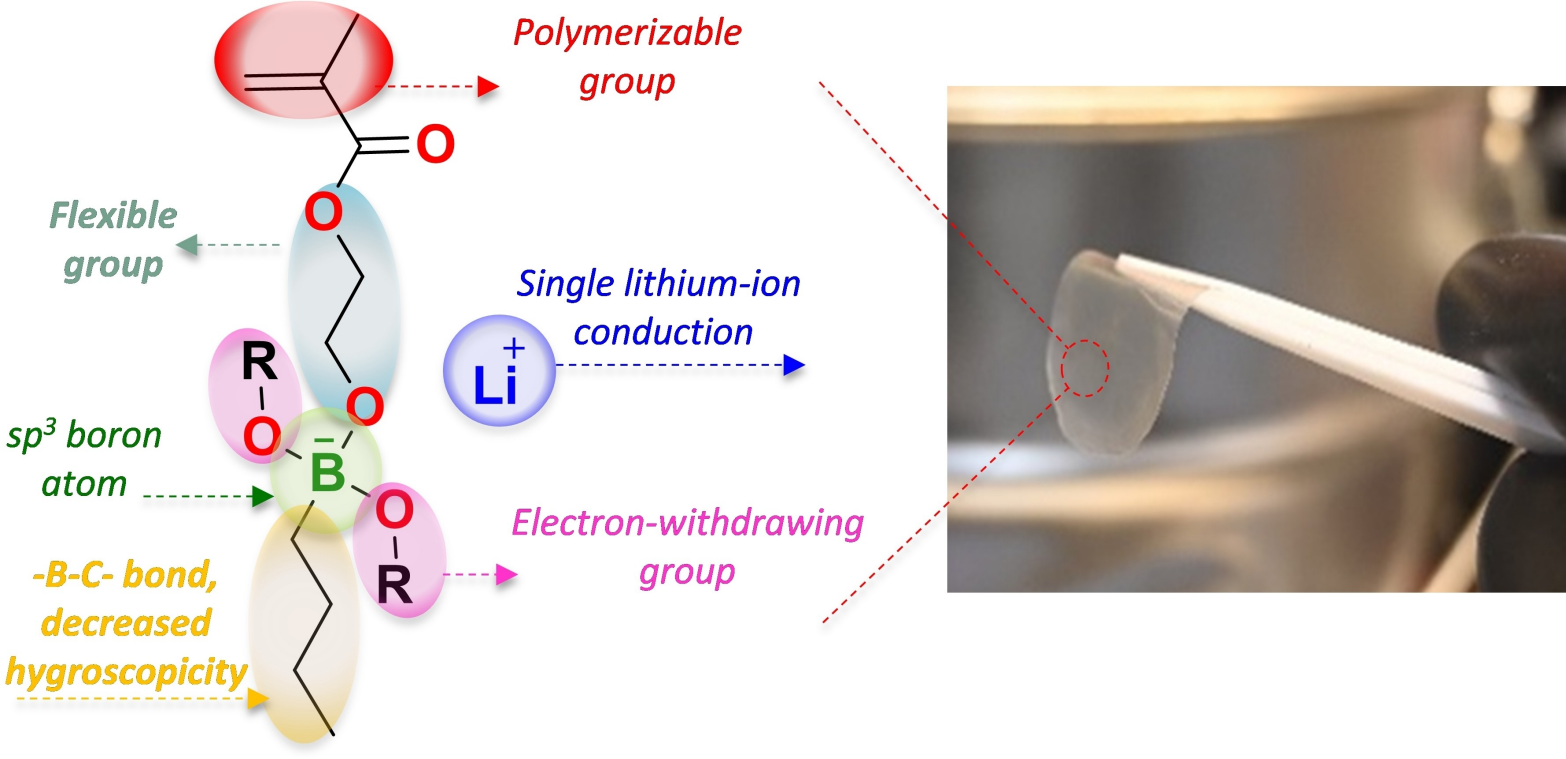
Design strategy for the borate lithium monomers and image of a homopolymer electrolyte membrane.

**Figure 2:**
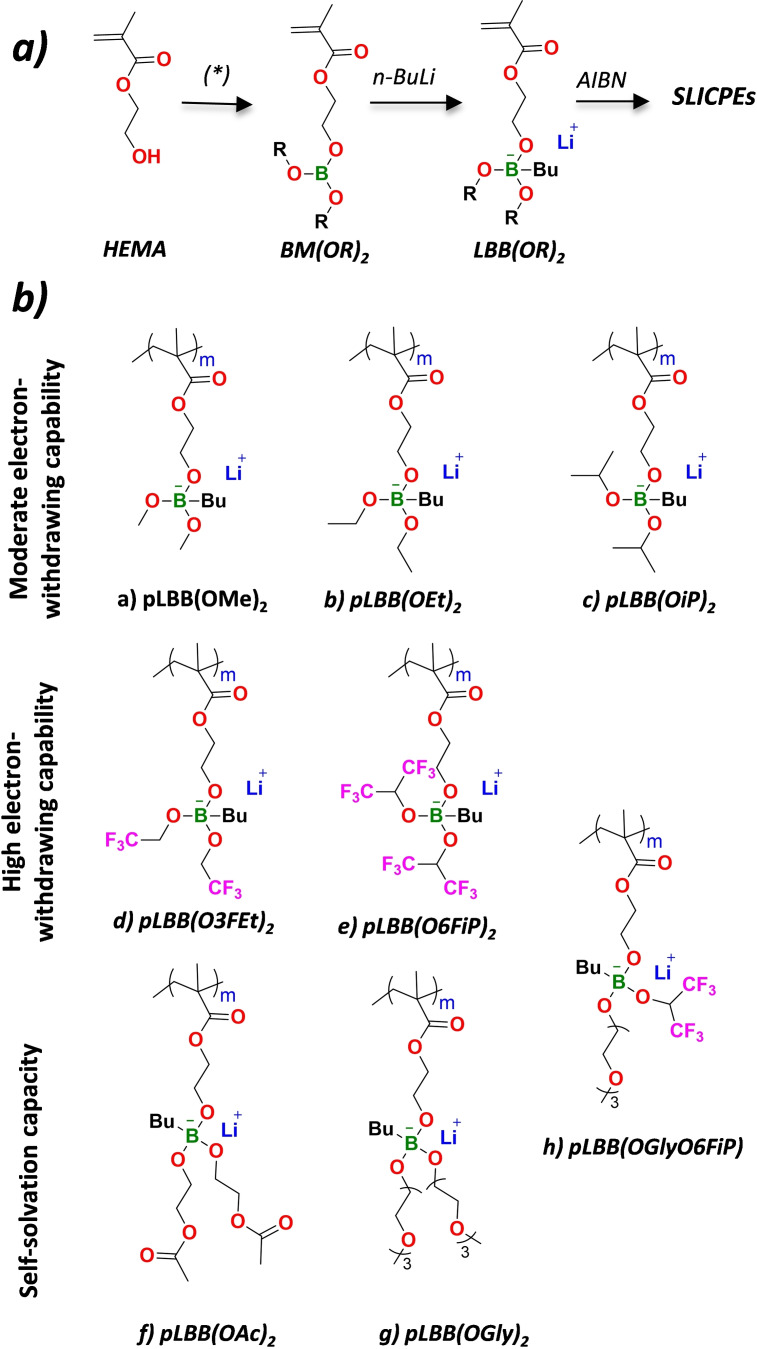
a) Synthetic route for the preparation of methacrylic lithium borate‐based monomers and b) chemical structures of SLICPEs, the yields for each of the reactions are included in the Supporting Information.

The ^1^H and ^11^B NMR spectra are presented to confirm the chemical structures of the monomers and polymers and to elucidate the effect of the different electron‐attracting groups on the electron density of the borate group. Figure 3 shows the spectra for pLBB(O6FiP)_2_, pLBB(OGlyO6FiP) and pLBB(OGly)_2_ polymers. The ^1^H NMR spectra (Figure 3a), in the region of 0.5 to 1.6 ppm present the signals associated with the methylene and methyl product of the polymerization of the methacrylate group in addition to the signals of the butyl group bonded to the boron atom. The ratio of these signals to the rest of the signals in each of the spectra decreases as a function of the ethoxy groups of the oligomeric chains whose protons are located in the region of 3.1 to 3.7 ppm, which in the case of the polymers with ethylene glycol chains overlap with the signals of the protons of the CH_2_ groups of the ethoxy ethyl methacrylate. The septet with a chemical shift at 4.5 ppm for pLBB(O6FiP)_2_ is associated with the unprotected carbon‐alpha proton of the (1,1,1,3,3,3‐hexafluoropropan‐2‐yl)oxy, which shows a decrease in intensity and a slight shift to high field (4.35 ppm) due to the effect of the addition of (methoxy tetraethylene glycol) as a substituent in the SLICPE pLBB(OGlyO6FiP).

**Figure 3:**
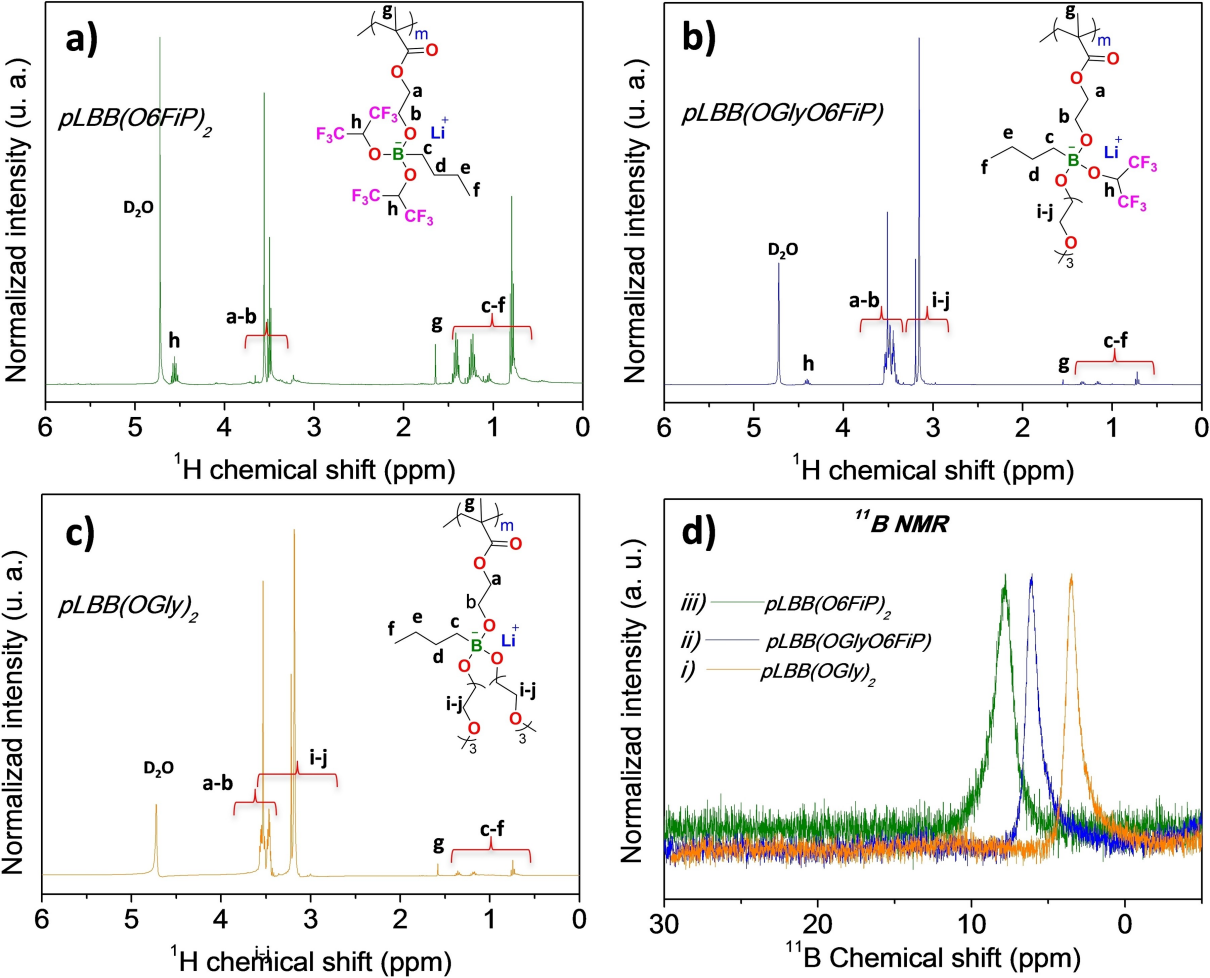
^1^H NMR spectra in D_2_O for synthesized SLICPEs: a) pLBB(O6FiP)_2_, b) pLBB(OGlyO6FiP), and c) pLBB(OGly)_2_, and d) ^1^B NMR spectra for these SLICPEs i–iii, respectively.

The ^11^B NMR spectra for the SLICPEs pLBB(O6FiP)_2_, pLBB(OGlyO6FiP), and pLBB(OGly)_2_ present a unique signal associated with the tetracoordinated boron atoms,^[23]^ with chemical shifts of 8, 6 and 4 ppm respectively, where the high chemical shift implies a higher degree of deprotection and decrease of the electron density of the central boron atom of the anionic groups, clearly influenced by the electron‐withdrawing capacity of substituent groups. This corroborates that the incorporation of fluorinated groups as substituents promotes the delocalization of the anionic charge of the boron atom. ^19^F NMR spectra showed the high purity of the pLBB(O6FiP)_2_ and pLBB(OGlyO6FiP) compounds (Figure S1).

The temperature dependence of the ionic conductivity of the borate polymers is shown in Figure 4. To easily understand the effect of the different substituents on the ionic conduction properties, the results will be discussed in three groups of borate polymers created according to the chemical characteristics of the substituents such as aliphatic, fluorinated, and self‐solvating.[^4^, ^24^] First, polymers substituted with low molecular weight aliphatic groups methyl, ethyl, isopropyl (Figure 4a–c): pLBB(OMe)_2_, pLBB(OEt)_2_, and pLBB(OiP)_2_ showed very similar and low ionic conductivity values of 3.29×10^−8^, 4.42×10^−9^, and 7.78×10^−9^ S cm^−1^ at 25 °C, respectively. It is observed that ionic conductivity at low temperature is almost not affected by the size of the substituent aliphatic groups. This behavior is maintained as a function of temperature for pLBB(OMe)_2_ and pLiBB(OEt)_2_. However, pLBB(OiP)_2_ presents greater temperature dependence and its ionic conductivity value is slightly higher than those found for pLBB(OMe)_2_, probably due to the generation of conduction spaces and pathways, as a result of the movement of the larger substituent groups.^[25]^

**Figure 4:**
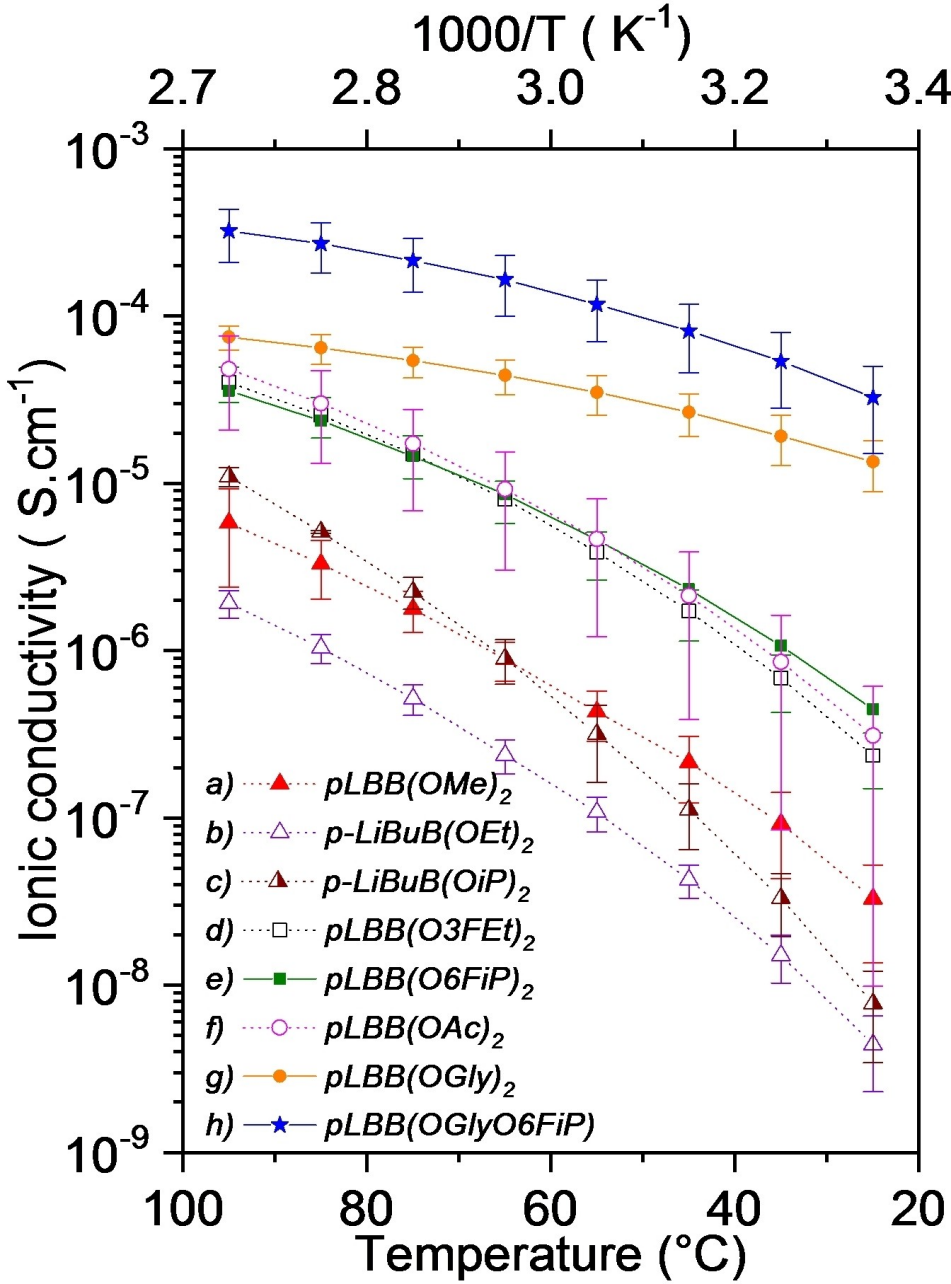
Temperature dependence of ionic conductivity for SLICPEs Boron‐based with several oxy‐substituents (pLBB(OR)_2_). Aliphatic groups: a) pLBB(OMe)_2_, b) pLBB(OEt)_2_, c) pLBB(OiP)_2_; fluorinated groups: d) pLBB(O3FEt)_2_, e) pLBB(O6FiP)_2_; solvating groups: f) pLBB(OGly)_2_, g) pLBB(OAc)_2_, and h) pLBB(OGlyO6FiP).

Second, polymers having borates with fluorinated groups as electron‐withdrawing substituents show a significant increase in the ionic conductivity values (Figure 4d,e). This ionic conductivity increase was previously observed for fluorinated cross‐linked polymer electrolytes.^[22]^ The ionic conductivity values were 2.36×10^−7^ and 4.46×10^−7^ S cm^−1^ at 25 °C for pLBB(O3FEt)_2_ and pLBB(O6FiP)_2_ SLICPEs, respectively. At low temperatures, the increase of the number of fluorine atoms in the electron‐withdrawing substituent groups generates a higher electronic delocalization in the anionic group, which increases the Li‐ions mobility. However, at high temperatures, the sum of thermal and electronic delocalization effects that contribute to their ionic conduction processes are comparable since they present similar ionic conductivity values, 8.01×10^−6^ and 8.61×10^−6^ S cm^−1^ at 60 °C for pLBB(O3FEt)_2_ and pLBB(O6FiP)_2_ polymers, respectively.

Third, the pLBB(OAc)_2_ polymer (Figure 4f), whose substituent “ethoxy acetate” groups provide self‐solvating effect,^[24]^ exhibits similar characteristics of magnitude and temperature dependence to polymers with fluorinated substituents. While the SLICPE pLBB(OGly)_2_ (Figure 4h) which includes two ethoxy chains as substituent groups with an O/Li^+^ ratio of 11, designed and synthesized to promote the self‐solvating effect of borate groups and provide pathways for ionic conduction, exhibited ionic conductivity values of 1.34×10^−5^, and 4.42×10^−5^ S cm^−1^ at 25 and 60 °C, respectively. The highest ionic conductivity results were obtained for the pLBB(OGlyO6FiP) homopolymer which combines a fluorinated substituent and an ethylene glycol one (Figure 4h), with also a significant increase in thermal stability (Figure S2). To our knowledge, the ionic conductivity values of 3.26×10^−5^ and 1.64×10^−4^ S cm^−1^ at 25 and 60 °C, respectively, are the highest reported for single Li‐ion conducting homopolymers.[^10^, ^14^, ^26^] This polymer combines asymmetric substituents in its molecular structure, thus the electronic delocalization associated with the O6FiP group, which allows one to decrease the interaction energy between the Li^+^‐borate group, and the incorporation of ethylene glycol chains as ion conduction pathways, generating a synergistic effect that provides improved conditions for ionic transport. In highly conductive SLICPEs, ionic transport is determined by the degree of super‐delocalized negative charge distribution of the anionic centers, which decreases the interaction energy in the ionic pair.^[10]^

The glass transition *T*_g_ values were measured for the SLICPEs pLBB(O6FiP)2, pLBB(OGlyO6FiP), and pLBB(OGly)_2_ showing values of −30 °C, −55 °C and −62 °C, respectively (Figure S3). Pseudo activation energy values calculated for the ionic conduction processes by the VTF equation[^27^, ^28^] of 0.47, 0.24 and 0.17 eV were obtained for the SLICPEs pLBB(O6FiP)_2_, pLBB(OGlyO6FiP), and pLBB(Ogly)_2_, respectively (Figure S4). These values confirm that the Li^+^ mobility is preferentially through the flexible ethoxide groups of the monomers, while the ionic conduction mechanisms in the fluorinated SLICPEs occurs preferentially by hopping through the interchain or active sites of the polymeric matrix.^[25]^

Furthermore, generally accepted models for Li^+^ transport in SPEs involving coupling to the segmental motion of the polymer backbone, a more flexible backbone is naturally beneficial for conductivity.^[6]^ Both assumptions for the description of ionic transport in SPE are synergically combined in the molecular structure of the pLBB(OGlyO6FiP) polymer, resulting in high ionic conductivity values.^[4]^ In order to verify the lithium single‐ion conducting characteristics the lithium transference number was measured. As expected, the optimized pLBB(OGlyO6FiP) homopolymer presents *t* ${}_{\mathrm{Li}{}^{+}}$ values of 0.93 (Figure S5). Furthermore, the homopolymer shows an electrochemical stability of 4.2 V vs. Li^0^/Li^+^ (Figure S6) confirming its excellent properties as SLIPCE for batteries.

The versatility of the methacrylic chemistry should allow one to use these monomers in different polymer formulations such as gel cross‐linked networks, random or block copolymers.[^2^, ^15^, ^17^, ^18^, ^19^] This should lead to an improvement in its ionic conductivity values. To prove this, a single‐ion gel cross‐linked polymer electrolyte based on LBB(OGlyO6FiP) monomer was formulated by rapid UV photopolymerization in the presence of a PEG‐diacrylate crosslinker (20 %wt.) and tetra‐glyme (60 %wt.) as plasticizer, and named as (GPE‐BB) (Figure 5a). This GPE‐BB gel polymer electrolyte showed an ionic conductivity value of 6.2×10^−4^ S cm^−1^ at 60 °C together with a high lithium transference number (*t* ${}_{\mathrm{Li}{}^{+}}$ =0.85). This gel electrolyte was mounted in a lithium‐metal symmetrical cell to evaluate its polarization resistance at different current densities ±0.01, ±0.1, ±0.2, ±0.5 mA cm^−2^ subsequently at 60 °C (Figure 5b). The critical current density (CCD) was ±0.2 mA cm^−2^ for cell, achieving overpotentials of <0.34 vs. Li^0^/Li^+^. Finally, the stability during long‐term cycling was performed after thermal conditioning at 50 °C for 3 h under OCV conditions. The Li^0^/GPE‐BB/Li^0^ cell was cycling at a current density value of ±0.2 mA cm^−2^ for 175 h (Figure 5c). The fact that the values of polarization potentials <0.85 V vs. Li^0^/Li^+^ remain constant during the whole test revealed a high electrochemical stability, so it is considered that the synthesized GPE‐BBs possess the necessary characteristics to be used in the optimized formulation of electrolytes for LIBs. The GPE‐BB could be further optimized by increasing the amount and characteristics of the plasticizers. This may improve the electrochemical stability of the GPE (Figure S7). Likewise, the use of other plasticizers could be the key to improving the electrochemical performance. For example, results on the use of diglyme instead of tetraglyme are shown in Figure 5b. Finally, Figure S9 shows an initial discharge of lithium–oxygen batteries (Figure S9), which reveals that the borate single‐ion GPES could be used in a full cell.

**Figure 5:**
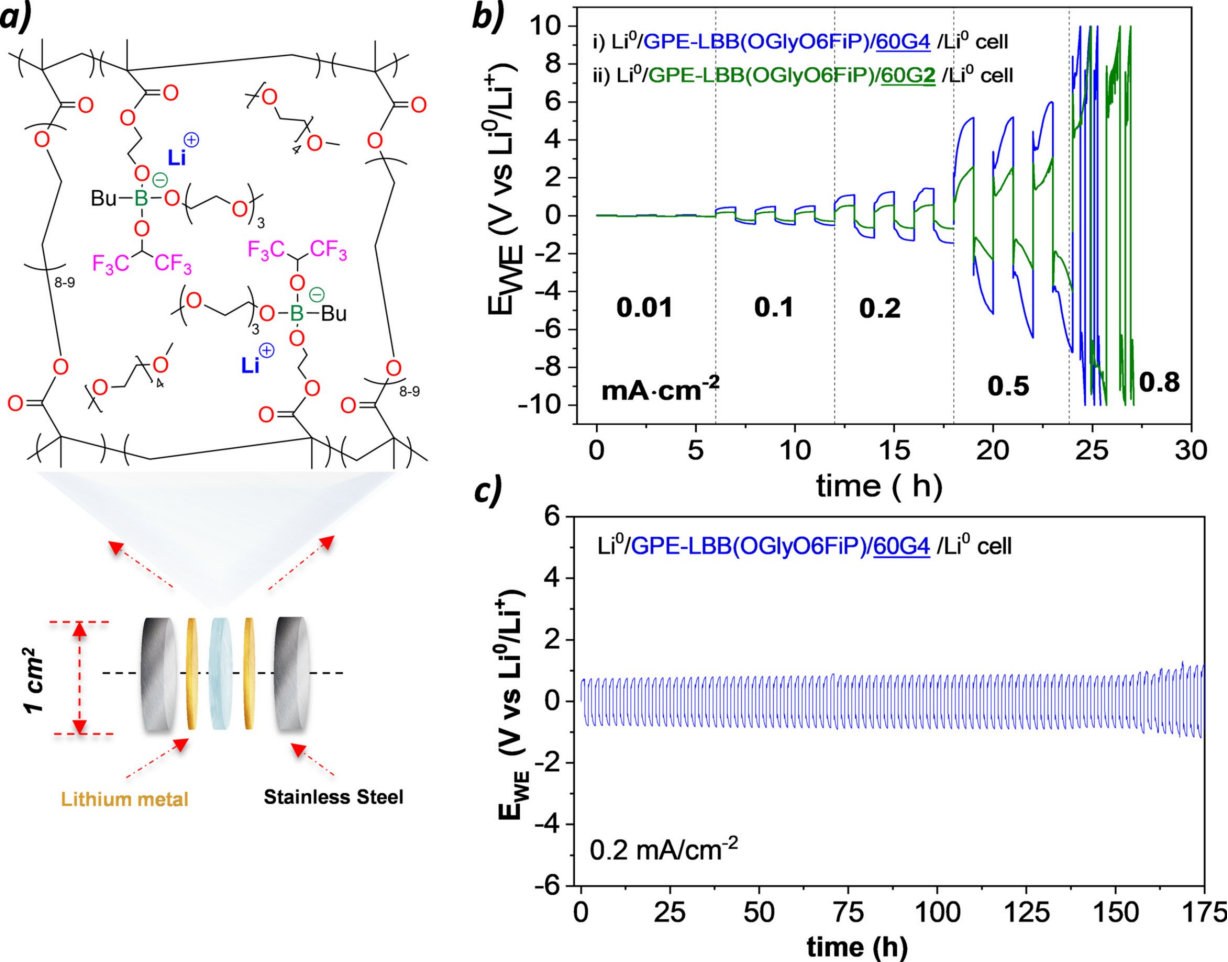
GPE based on LBB(OGlyO6FiP): a) concept scheme, b) polarization resistance at different current densities c) Li^+^ plating/stripping curves of Li^0^/GPE‐BB/Li^0^ symmetric cell at a current density of 0.2 mA cm^−2^.

In summary, we report here a new family of single‐ion lithium conducting methacrylic polymer electrolytes based on highly delocalized borate groups. The effect of the nature of the substituents on the boron atom including aliphatic, fluorinated, and self‐solvating on their ionic conductivity was analyzed. The optimized methacrylic borate SLIPCE shows, to the best of our knowledge, the highest ionic conductivity reported for a lithium single‐ion conduction homopolymer (1.65×10^−4^ S cm^−1^ at 60 °C). The single‐ion conducting properties were confirmed by its high *t* ${}_{\mathrm{Li}{}^{+}}$ =0.93. The versatility of the acrylic polymer chemistry to include this monomer in future (co)polymer and polymer electrolytes formulations will be explored together with its application in lithium batteries.

## Conflict of interest

The authors declare no conflict of interest.

## Supporting information

As a service to our authors and readers, this journal provides supporting information supplied by the authors. Such materials are peer reviewed and may be re‐organized for online delivery, but are not copy‐edited or typeset. Technical support issues arising from supporting information (other than missing files) should be addressed to the authors.

Supporting Information
