# Supplementary material for: Single‐Ion Lithium Conducting Polymers with High Ionic Conductivity Based on Borate Pendant Groups
Source: Angew Chem Int Ed Engl. 2021 Dec 29;61(7):e202114024. doi: 10.1002/anie.202114024 (PMC9306921; doi:10.1002/anie.202114024)
Supplement: Supplementary file 1 — Supporting Information [file ANIE-61-0-s001.pdf]

Angewandte Chemie
Eine Zeitschrift der Gesellschaft Deutscher Chemiker

Supporting Information

Single-Ion Lithium Conducting Polymers with High Ionic Conductivity Based on Borate Pendant Groups

G. Guzmán-González, S. Vauthier, M. Alvarez-Tirado, S. Cotte, L. Castro, A. Guéguen, N. Casado, D. Mecerreyes**

Table of Contents

I. *Materials characterization and electrochemical measurements* (2)
II. *Experimental* (2)
a. *Synthesis of lithium butyl(2-(boryl)oxy)ethyl methacrylate monomers* (2)
b. *Polymerization procedure* (4)
III. *Additional figures* (5)
Figure S1: ^{19}F NMR spectra of SLICPEs (5)
Figure S2: TGA curves of SLICPEs (6)
Figure S3: DSC curves of SLICPEs (6)
Figure S4: Ionic conductivities as a function of temperature for selected SLICPEs and their fit to the VTF equation (7)
Figure S5: Lithium transference number measurements for SLICPEs (7)
Figure S6: Electrochemical stability windows of homopolymer pLBB(OGlyO6FiP) (8)
Figure S7: Electrochemical stability windows of GPE LBB(OGlyO$_6$FiP)/60G4 and LBB(OFiP)$_2$/60G4 (8)
Figure S8: Polarization resistance at different current densities for GPE-BB (9)
Figure S9: Li-O_2 cells using pLBB(OGlyO6FiP)/60G4 as an electrolyte (preliminary results) (9)
IV. *Author Contributions* (9)

Experimental Procedures

I. Materials characterization and electrochemical measurements

^1H nuclear magnetic resonance spectra (Avance III 400 MHz Digital NMR spectrometer) and Fourier transform infrared spectra (Nicolet 6700 FTIR spectrometer over the range of 4000–400 cm^{-1}) were used to characterize structure information for the monomers and SLICPEs based boron. The thermal properties of the polymers were evaluated by thermogravimetric analysis (TGA, SDTQ-600 TA), with a heating rate of 10 °C min^{-1} from 30° to 650°C under an N_2 atmosphere, and differential scanning calorimetry (DSC, SDTQ-600 TA) with a heating rate of 10 °C min^{-1} from −70 to 100 °C under N_2 flow.
Ionic conductivity for SLICPEs was triplicate by electrochemical impedance spectroscopy (EIS) using an Autolab 302N potentiostat/ galvanostat (Metrohm AG) at different temperatures (100–25 °C), equipped with a temperature controller (Microcell HC station). The sample was placed between two stainless steel electrodes (surface area = 0.5 cm^2). The impedance spectrum was performed open-circuit conditions applying a perturbation voltage of 10 mV in over a frequency range of 10^5 - 1 Hz. The electrochemical stability was evaluated with a stainless steel/SLICPE/Li coin cell using linear sweep voltammetry (LSV) in a range from 2.5 to 5 V vs Li^0/Li^+ at a scan rate of 2 mV s^{-1} at 25 °C. The direct current (DC) polarization/alternating current (AC) impedance method was employed to evaluate the lithium-ion transference number (t_{Li^+}) in a Li^0/SLICPEs/Li^0 symmetric coin cell, by P. Bruce Method.

II. Experimental

Chemicals. — 2-Hydroxyethyl methacrylate (HEMA, 97%, Aldrich), Trimethyl borate (TMB, ≥98%, Aldrich), Triethyl borate (TEB, ≥95%, Aldrich), Triisopropyl borate (TiPB, ≥98%, Aldrich), Borane tetrahydrofuran complex solution 1.0 M in THF (BH_3, Aldrich), 2,2,2-Trifluoroethanol (TFE, Aldrich), 1,1,1,3,3,3-Hexafluoro-2-propanol (HFiP, ≥99%, Aldrich), n-Butyllithium 2.5M solution in hexanes, (nBuLi, ACROS), and 2,2´-Azobisisobutyronitrile (AIBN, initiator, 98%, Aldrich) were used as received. 2-Hydroxyethyl Acetate (HEA, 75%, TCI), and Triethylene glycol monomethyl ether (TEG, 95%, Aldrich) were distilled at the rotary evaporator at 70°C and reduced pressure. The solvents Methanol and Hexane from SharpLab were dried with anhydrous $MgSO_4$ before used.

Synthesis of lithium butyl(2-(boryl)oxy)ethyl methacrylate monomers.
Method A.
Lithium butyl(2-((dimethoxyboryl)oxy)ethyl methacrylate) ***(LBB(OMe)$_2$)***
2-Hydroxyethyl methacrylate (10 mmol, 1.3 g) and dry hexane (30 ml) were charged into a flask of 100 ml, the solution was stirred with argon flow and then cooled in an acetone-liquid nitrogen bath, avoiding solidification of the system. Then Trimethyl borate (10 mmol, 1.04 ml) was added dropwise, the reaction mixture was slowly heated to room temperature (RT) and stirred for 2 h. Subsequently, the system was cooled again in an acetone-liquid nitrogen bath and dropwise n-Butyl lithium 2.5 M in hexane (10 mmol, 4 ml) was added. The precipitate formed was heated to RT and stirred for a further 2 h more, before being filtered and washed with cold diethyl ether.

The white powder obtained was placed in a vial and dried on a vacuum line at 40°C for 24h. Yield: 2.25 g (87%); Found: ^{1}H NMR (400 MHz, D_2O): δ (ppm) = 6.02 (s, 1H, $\underline{CH_2}$=C(CH_3)–), 5.51 (t, 1H, $\underline{CH_2}$=C(CH3)–), 4.19 (t, 2H, CO-O-$\underline{CH_2}$), 3.86 (t, 2H, CO-O-CH_2-$\underline{CH_2}$-O-B), 3.39 (s, 6H, B-O-$\underline{CH_3}$), 1.85 (s, 3H, CH_2-C($\underline{CH_3}$)); 1.41 (q, 4H, B-$\underline{CH_2}$-CH_2-); 1.22 (sx, 4H, B-CH_2-$\underline{CH_2}$-); 1.15 (t, 12H, B-$\underline{CH_2}$); 0.78 (c, 4H, $(CH_2)_2$-$\underline{CH_3}$); ^{11}B NMR (400 MHz, D_2O): δ (ppm) = 8.2 (s, -CH_2-$\underline{B}$-$(OR)_2$);

Lithium butyl 2-((diethoxyboryl)oxy)ethyl methacrylate) ***(LBB(OEt)₂)***

2-Hydroxyethyl methacrylate (10 mmol, 1.3 g) and dry hexane (30 ml) were charged into a flask of 100 ml, the solution was stirred with argon flow and then cooled in an acetone-liquid nitrogen bath, avoiding solidification of the system. Then Tri-ethyl borate (10 mmol, 1.7 ml) was added dropwise, the reaction mixture was slowly heated to RT and stirred for 2 h. Subsequently, the system was cooled again in an acetone-liquid nitrogen bath and dropwise n-Butyl lithium 2.5 M in hexane (10 mmol, 4 ml) was added. The precipitate formed was heated to RT and stirred for a further 2 h more, before being filtered and washed with cold diethyl ether. The white powder obtained was placed in a vial and dried on a vacuum line at 40°C for 24h. Yield: 2.67 g (93%); Found: ^{1}H NMR (400 MHz, D_2O): δ (ppm) = 6.02 (s, 1H, $\underline{CH_2}$=C(CH_3)–), 5.51 (t, 1H, $\underline{CH_2}$=C(CH3)–), 4.19 (t, 2H, CO-O-$\underline{CH_2}$), 3.86 (t, 2H, CO-O-CH_2-$\underline{CH_2}$-O-B), 3.91 (c, 6H, B-O-$\underline{CH_2}$), 1.85 (s, 3H, CH_2-C($\underline{CH_3}$)); 1.41 (q, 4H, B-$\underline{CH_2}$-CH_2-); 1.22 (sx, 4H, B-CH_2-$\underline{CH_2}$-); 1.15 (t, 12H, B-$\underline{CH_2}$); 1.09 (t, 6H, B-O-CH_2-$\underline{CH_3}$), 0.78 (c, 4H, $(CH_2)_2$-$\underline{CH_3}$); ^{11}B NMR (400 MHz, D_2O): δ (ppm) = 8.2 (s, -CH_2-$\underline{B}$-$(OR)_2$).

*Lithium butyl(2-((diisopropoxyboryl)oxy)ethyl methacrylate) (**LBB(OiP)₂**).*

2-Hydroxyethyl methacrylate (10 mmol, 1.3 g) and dry hexane (30 ml) were charged into a flask of 100 ml, the solution was stirred with argon flow and then cooled in an acetone-liquid nitrogen bath, avoiding solidification of the system. Then Tri-isopropyl borate (10 mmol, 2.2 ml) was added dropwise, the reaction mixture was slowly heated to RT and stirred for 2 h. Subsequently, the system was cooled again in an acetone-liquid nitrogen bath and dropwise n-Butyl lithium 2.5 M in hexane (10 mmol, 4 ml) was added. The precipitate formed was heated to RT and stirred for a further 2 h more, before being filtered and washed with cold diethyl ether. The white powder obtained was placed in a vial and dried on a vacuum line at 40°C for 24h. Yield: 2.99 g (95 %); Found: ^{1}H NMR (400 MHz, D_2O): δ (ppm) = 6.02 (s, 1H, $\underline{CH_2}$=C(CH_3)–), 5.51 (t, 1H, $\underline{CH_2}$=C(CH3)–), 4.19 (t, 2H, CO-O-$\underline{CH_2}$), 3.86 (t, 2H, CO-O-CH_2-$\underline{CH_2}$-O-B), 3.57 (m, 2H, B-O-$\underline{C}$$(CH_3)_2$); 1.85 (s, 3H, CH_2-C($\underline{CH_3}$)); 1.41 (q, 4H, B-$\underline{CH_2}$-CH_2-); 1.22 (sx, 4H, B-CH_2-$\underline{CH_2}$-); 1.15 (t, 12H, B-$\underline{CH_2}$); 1.13 (d, 12H, B-O-C$(\underline{CH_3})_2$); 0.78 (c, 4H, $(CH_2)_2$-$\underline{CH_3}$); ^{11}B NMR (400 MHz, D_2O): δ (ppm) = 8.2 (s, -CH_2-$\underline{B}$-$(OR)_2$).

Method B

Lithium butyl 2-((bis(2,2,2-trifluoroethoxy)boryl)oxy)ethyl methacrylate (LBB(O₃FEt)₂).

2-Hydroxyethyl methacrylate (10 mmol, 1.3 g) and 30 ml of dry hexane were charged into the 100 ml two-neck flask, the solution was stirred with argon flow and subsequently cooled in an acetone-liquid N_2 bath, avoiding solidification of the system. BH3-THF complex solution 1M in THF (10 mmol, 10 ml) was carefully added "dropwise", while H_2 was expelled from the system, then the reaction mixture was slowly warmed to room temperature and stirred for 30 min more. Subsequently, the system was cooled again in an acetone-liquid N_2 bath and 2,2,2,2-Trifluoroethanol (20 mmol, 4 ml) was dropwise added, the system was again heated to RT. for 1 hour to ensure the second evolution of H_2 has ended. Then the system was again cooled in an acetone-liquid N_2 bath and carefully added n-Butyl lithium 2.5 M in hexane (10 mmol, 4 ml). A transparent gel was formed, which was stirred at room temperature for a further 2 hours. Finally, the product was precipitated and washed with cold diethyl ether. The obtained clear gel was placed in a vial and dried in a vacuum line at 40°C for 24h. Found: Yield: 3.36 g (85%); ^{1}H NMR (400 MHz, D_2O): δ (ppm) = 6.02 (s, 1H, $\underline{CH_2}$=C(CH_3)–), 5.51 (t, 1H, $\underline{CH_2}$=C(CH3)–), 4.38 (c, 2H, B-O-$\underline{CH_2}$$CF_3$); 4.19 (t, 2H, CO-O-$\underline{CH_2}$), 3.86 (t, 2H, CO-O-$CH_2$-$\underline{CH_2}$-O-B), 1.85 (s, 3H, CH_2-C($\underline{CH_3}$)); 1.39 (q, 4H, B-$\underline{CH_2}$-CH_2-); 1.23 (sx, 4H, B-CH_2-$\underline{CH_2}$-); 1.03 (c, 4H, $(CH_2)_2$-$\underline{CH_3}$); 0.78 (t, 12H, B-$\underline{CH_2}$); ^{11}B NMR (400 MHz, D_2O): δ (ppm) = 8.2 (s, -CH_2-$\underline{B}$-$(OR)_2$); ^{19}F NMR (400 MHz, D_2O): δ (ppm) = -78.2 (s, CF_3).

*Lithium butyl(2-((bis((1,1,1,3,3,3-hexafluoropropan-2-yl)oxy)boryl)oxy)ethyl methacrylate) (**LBB(O6FiP)₂).***

2-Hydroxyethyl methacrylate (10 mmol, 1.3 g) and 30 ml of dry hexane were charged into the 100 ml two-neck flask, the solution was stirred with argon flow and subsequently cooled in an acetone-liquid N_2 bath, avoiding solidification of the system. BH3-THF complex solution 1M in THF (10 mmol, 10 ml) was carefully added "dropwise", while H_2 was expelled from the system, then the reaction mixture was slowly warmed to room temperature and stirred for 30 min more. Subsequently, the system was cooled again in an acetone/liquid N_2 bath, and 1,1,1,3,3,3-Hexafluoro-2-propanol (20 mmol, 4 ml) was dropwise added, the system was again heated to RT. for 1 hour to ensure the second evolution of H_2 has ended. Then the system was again cooled in an acetone-liquid N_2 bath and carefully added n-Butyl lithium 2.5 M in hexane (10 mmol, 4 ml). A viscous transparent liquid was formed, which was stirred at room temperature for a further 2 hours. Finally, the product was precipitated and washed with cold diethyl ether. The obtained transparent liquid was placed in a vial and dried in a vacuum line at 40°C for 24h. Yield: 4.62 g (87%); Found: ^{1}H NMR (400 MHz, D_2O): δ (ppm) = 6.02 (s, 1H, $\underline{CH_2}$=C(CH_3)–), 5.51 (t, 1H, $\underline{CH_2}$=C(CH_3)–), 4.38 (sp, 2H, B-O-$\underline{CH}$$(CF_3)_2$); 4.19 (t, 2H, CO-O-$\underline{CH_2}$), 3.86 (t, 2H, CO-O-$CH_2$-$\underline{CH_2}$-O-B), 1.85 (s, 3H, CH_2-C($\underline{CH_3}$)); 1.39 (q, 4H, B-$\underline{CH_2}$-CH_2-); 1.23 (sx, 4H, B-CH_2-$\underline{CH_2}$-); 1.03 (c, 4H, $(CH_2)_2$-$\underline{CH_3}$); 0.78 (t, 12H, B-$\underline{CH_2}$); ^{11}B NMR (400 MHz, D_2O): δ (ppm) = 8.2 (s, -CH_2-$\underline{B}$-$(OR)_2$); ^{19}F NMR (400 MHz, D_2O): δ (ppm) = -78.2 (s, CF_3).

*Lithium butyl(2-((bis(ethane-2,1-diyl) diacetate)boryl)oxy)ethyl methacrylate) (**LBB(OAc)₂**).*

2-Hydroxyethyl methacrylate (10 mmol, 1.3 g) and 30 ml of dry hexane were charged into the 100 ml two-neck flask, the solution was stirred with argon flow and subsequently cooled in an acetone-liquid N_2 bath, avoiding solidification of the system. BH3-THF complex solution 1M in THF (10 mmol, 10 ml) was carefully added "dropwise", while H_2 was expelled from the system, then the reaction mixture

was slowly warmed to room temperature and stirred for 30 min more. Subsequently, the system was cooled again in an acetone/liquid N_2 bath and 2-Hydroxyethyl Acetate (20 mmol, 4 ml) was dropwise added, the system was again heated to RT. for 1 hour to ensure the second evolution of H_2 has ended. Then the system was again cooled in an acetone-liquid N_2 bath and carefully added n-Butyl lithium 2.5 M in hexane (10 mmol, 4 ml). A viscous transparent liquid was formed, which was stirred at room temperature for a further 2 hours. Finally, the product was precipitated and washed with cold diethyl ether. The obtained transparent liquid was placed in a vial and dried in a vacuum line at 40°C for 24h. Yield: 3.22 g (80 %); Found: ^{1}H NMR (400 MHz, D_2O): δ (ppm) = 6.02 (s, 1H, $\underline{CH_2}$=C(CH_3)–), 5.51 (t, 1H, $\underline{CH_2}$=C(CH_3)–), 4.19 (t, 2H, CO-O-$\underline{CH_2}$), 4.38 (t, 4H, CO-O-$\underline{CH_2}$); 3.86 (t, 2H, CO-O-CH_2-$\underline{CH_2}$-O-B), 3.76 (t, 4H, CO-O-CH_2-$\underline{CH_2}$-O-B), 2.01 (s, 3H, CO-$\underline{CH_3}$); 1.85 (s, 3H, CH_2-C($\underline{CH_3}$)); 1.39 (q, 4H, B-$\underline{CH_2}$-CH_2-); 1.23 (sx, 4H, B-CH_2-$\underline{CH_2}$-); 1.03 (c, 4H, $(CH_2)_2$-$\underline{CH_3}$); 0.78 (t, 12H, B-$\underline{CH_2}$); ^{11}B NMR (400 MHz, D_2O): δ (ppm) = 8.2 (s, -CH_2-$\underline{B}$-$(OR)_2$).

Lithium butyl(2-((bis((2-(2-(2-methoxyethoxy)ethoxy)ethyl)boryl)oxy)ethyl methacrylate) ***(LBB(OGly)₂)***.

2-Hydroxyethyl methacrylate (10 mmol, 1.3 g) and 30 ml of dry hexane were charged into the 100 ml two-neck flask, the solution was stirred with argon flow and subsequently cooled in an acetone-liquid N_2 bath, avoiding solidification of the system. BH3-THF complex solution 1M in THF (10 mmol, 10 ml) was carefully added "dropwise", while H_2 was expelled from the system, then the reaction mixture was slowly warmed to room temperature and stirred for 30 min more. Subsequently, the system was cooled again in an acetone/liquid N_2 bath and Triethylene glycol monomethyl ether (20 mmol, 4 ml) was dropwise added, the system was again heated to RT. for 1 hour to ensure the second evolution of H_2 has ended. Then the system was again cooled in an acetone-liquid N_2 bath and carefully added n-Butyl lithium 2.5 M in hexane (10 mmol, 4 ml). A viscous transparent liquid was formed, which was stirred at room temperature for a further 2 hours. Finally, the product was precipitated and washed with cold diethyl ether. The obtained transparent liquid was placed in a vial and dried in a vacuum line at 40°C for 24h. Yield: 4.45 g (85%); Found: ^{1}H NMR (400 MHz, D_2O): δ (ppm) = 6.02 (s, 1H, $\underline{CH_2}$=C(CH_3)–), 5.51 (t, 1H, $\underline{CH_2}$=C(CH_3)–), 4.19 (t, 2H, CO-O-$\underline{CH_2}$), 3.86 (t, 2H, BO-$\underline{CH_2}$-CH_2-COO), 3.80-3.46 (m, 24H, O-$\underline{CH_2}$-$\underline{CH_2}$-O); 3.31 (s, 6H, CO-$\underline{CH_3}$); 1.85 (s, 3H, CH_2-C($\underline{CH_3}$)); 1.35 (q, 4H, B-$\underline{CH_2}$-CH_2-); 1.28 (sx, 4H, B-CH_2-$\underline{CH_2}$-); 0.21 (c, 4H, $(CH_2)_2$-$\underline{CH_3}$); 0.74 (t, 12H, B-$\underline{CH_2}$); ^{11}B NMR (400 MHz, D_2O): δ (ppm) = 8.2 (s, -CH_2-$\underline{B}$-$(OR)_2$).

Lithium butyl(2-((2-((1,1,1,3,3,3-hexafluoropropan-2-yl (2-(2-(2-methoxyethoxy)ethoxy) ethyl) boryl)oxy)ethyl methacrylate) ***(LBB(OGlyO6FiP)***.

2-Hydroxyethyl methacrylate (10 mmol, 1.3 g) and 30 ml of dry hexane were charged into the 100 ml two-neck flask, the solution was stirred with argon flow and subsequently cooled in an acetone-liquid N_2 bath, avoiding solidification of the system. BH3-THF complex solution 1M in THF (10 mmol, 10 ml) was carefully added "dropwise", while H_2 was expelled from the system, then the reaction mixture was slowly warmed to room temperature and stirred for 30 min more. Subsequently, the system was cooled again in an acetone/liquid N_2 bath, and Triethylene glycol monomethyl ether (10 mmol, 4 ml) was dropwise added, the system was again heated to RT. for 1 hour to ensure the second evolution of H_2 has ended. Then the system was cooled again in an acetone/liquid N_2 bath and 1,1,1,3,3,3-Hexafluoro-2-propanol (10 mmol, 4 ml) was dropwise added, the system was again heated to RT. for 1 hour to ensure the third evolution of H_2 has ended. Then the system was again cooled in an acetone-liquid N_2 bath and carefully added n-BuLi 2.5 M in hexane (10 mmol, 4 ml). A viscous transparent liquid was formed, which was stirred at room temperature for a further 2 hours. Finally, the product was precipitated and washed with cold diethyl ether. The obtained transparent liquid was placed in a vial and dried in a vacuum line at 40°C for 24h. Yield: 4.85 g (0.92%); Found: ^{1}H NMR (400 MHz, D_2O): δ (ppm) = 6.02 (s, 1H, $\underline{CH_2}$=C(CH_3)–), 5.51 (t, 1H, $\underline{CH_2}$=C(CH_3)–), 4.38 (sp, 1H, B-O-$\underline{CH}$(CF_3)$_2$); 4.19 (t, 2H, CO-O-$\underline{CH_2}$), 3.86 (t, 2H, BO-$\underline{CH_2}$-CH_2-COO), 3.80-3.46 (m, 12H, O-$\underline{CH_2}$-$\underline{CH_2}$-O); 3.31 (s, 6H, CO-$\underline{CH_3}$); 1.85 (s, 3H, CH_2-C($\underline{CH_3}$)); 1.35 (q, 4H, B-$\underline{CH_2}$-CH_2-); 1.28 (sx, 4H, B-CH_2-$\underline{CH_2}$-); 0.21 (c, 4H, $(CH_2)_2$-$\underline{CH_3}$); 0.74 (t, 12H, B-$\underline{CH_2}$); ^{11}B NMR (400 MHz, D_2O): δ (ppm) = 8.2 (s, -CH_2-$\underline{B}$-$(OR)_2$); ^{19}F NMR (400 MHz, D_2O): δ (ppm) = -78.2 (s, CF_3).

Polymerization procedure

The synthesized boron-based monomers were used to obtain a series of SLICPEs using the random radical polymerization method. The following example describes the procedure used for the synthesis of the linear polymer pLBB$(OMe)_2$: LBB$(OMe)_2$ monomer (0.95 g), AIBN (0.0040 g, 3 wt%), and methanol (0.40 ml) were gently mixed in a Schlenk tube at room temperature. To remove as much oxygen as possible, the system was bubbled for 3 min with a flow of argon and an additional 30 min after the reagents were added. The reaction flask was then immersed in a hot oil bath at 60°C and left for 6h. After the reaction, the polymer was precipitated in cold diethyl ether. Finally, the polymer was thoroughly dried at 60 °C under a high vacuum for 24 h and stored in the glove box. The sintered monomers (*LBB(OR)₂*) were used for obtaining SLICPEs using the same polymerization method described above for obtaining (p*LBB(OR)₂*), and labeled according to the nomenclature of their precursor monomers as *pLBB(OMe)₂*, *pLBB(OEt)₂*, *pLBB(OiP)₂*, *pLBB(O3FEt)₂*, *pLBB(O6FiP)₂*, *pLBB(OGly)₂*, *pLBB(OAc)₂*, *and pLBB(OGlyO6FiP)*, respectively. The result of the structural characterization of these polymers is reported below:

***pLBB(OMe)₂*,** Yield: 0.9 g (97%); ^{1}H NMR (400 MHz, D_2O): δ (ppm) = 3.75-3.44 (dt, 2H, CO-O-$\underline{CH_2}$-$\underline{CH_2}$-O-B), 3.24 (s, 6H, B-O-$\underline{CH_3}$), 1.62 (s, 3H, CH_2-C($\underline{CH_3}$)-CH_2); 1.41 (q, 4H, B-$\underline{CH_2}$-CH_2-); 1.22 (sx, 4H, B-CH_2-$\underline{CH_2}$-); 1.15 (t, 12H, B-$\underline{CH_2}$); 0.78 (c, 4H, $(CH_2)_2$-$\underline{CH_3}$); ^{11}B NMR (400 MHz, D_2O): δ (ppm) = 8.2 (s, -CH_2-$\underline{B}$-$(OR)_2$).

***pLBB(OEt)₂*,** Yield: 1.25 g (91%); ^{1}H NMR (400 MHz, D_2O): δ (ppm) = 3.75-3.44 (dt, 2H, CO-O-$\underline{CH_2}$-$\underline{CH_2}$-O-B), 3.22 (c, 6H, B-O-$\underline{CH_2}$), 1.62 (s, 3H, CH_2-C($\underline{CH_3}$)-CH_2); 1.41 (q, 4H, B-$\underline{CH_2}$-CH_2-); 1.22 (sx, 4H, B-CH_2-$\underline{CH_2}$-); 1.15 (t, 12H, B-$\underline{CH_2}$); 1.06 (t, 6H, B-O-CH_2-$\underline{CH_3}$), 0.78 (c, 4H, $(CH_2)_2$-$\underline{CH_3}$); ^{11}B NMR (400 MHz, D_2O): δ (ppm) = 8.2 (s, -CH_2-$\underline{B}$-$(OR)_2$).

pLBB(OiP)$_2$, Yield: 1.08 g (87%); ^{1}H NMR (400 MHz, D_2O): δ (ppm) = 3.91 (m, 2H, B-O-C(CH_3)$_2$); 3.75-3.44 (dt, 2H, CO-O-CH_2-CH_2-O-B), 1. 62 (s, 3H, CH_2-C(CH_3)-CH_2); 1.41 (q, 4H, B-CH_2-CH_2-); 1.22 (sx, 4H, B-CH_2-CH_2-); 1.15 (t, 12H, B-CH_2); 1.06 (d, 12H, B-O-C(CH_3)$_2$); 0.78 (c, 4H, (CH_2)$_2$-CH_3); ^{11}B NMR (400 MHz, D_2O): δ (ppm) = 8.2 (s, -CH_2-B-(OR)$_2$).

pLBB(O3FEt)$_2$, Yield: 1.05 g (82%); ^{1}H NMR (400 MHz, D_2O): δ (ppm) = 3.87 (c, 2H, B-O-CH_2CF_3); 3.75-3.44 (dt, 2H, CO-O-CH_2-CH_2-O-B), 1.62 (s, 3H, CH_2-C(CH_3)-CH_2); 1.39 (q, 4H, B-CH_2-CH_2-); 1.23 (sx, 4H, B-CH_2-CH_2-); 1.03 (c, 4H, (CH_2)$_2$-CH_3); 0.78 (t, 12H, B-CH_2); ^{11}B NMR (400 MHz, D_2O): δ (ppm) = 8.2 (s, -CH_2-B-(OR)$_2$); ^{19}F NMR (400 MHz, D_2O): δ (ppm) = -78.2 (s, CF_3).

pLBB(O6FiP)$_2$, Yield: 1.32 g (91%); ^{1}H NMR (400 MHz, D_2O): δ (ppm) = 4.46 (sp, 2H, B-O-CH(CF_3)$_2$); 3.75-3.44 (dt, 2H, CO-O-CH_2-CH_2-O-B), 1.62 (s, 3H, CH_2-C(CH_3)-CH_2); 1.39 (q, 4H, B-CH_2-CH_2-); 1.23 (sx, 4H, B-CH_2-CH_2-); 1.03 (c, 4H, (CH_2)$_2$-CH_3); 0.78 (t, 12H, B-CH_2); ^{11}B NMR (400 MHz, D_2O): δ (ppm) = 8.2 (s, -CH_2-B-(OR)$_2$); ^{19}F NMR (400 MHz, D_2O): δ (ppm) = -78.2 (s, CF_3).

pLBB(OAc)$_2$, Yield: 1.42 g (71%); ^{1}H NMR (400 MHz, D_2O): δ (ppm) = 3.79-3.38 (m, 12H, O-CH_2-CH_2-O-), 1.78 (s, 3H, CO-CH_3); 1.62 (s, 3H, CH_2-C(CH_3)-CH_2); 1.39 (q, 4H, B-CH_2-CH_2-); 1.23 (sx, 4H, B-CH_2-CH_2-); 1.03 (c, 4H, (CH_2)$_2$-CH_3); 0.78 (t, 12H, B-CH_2); ^{11}B NMR (400 MHz, D_2O): δ (ppm) = 8.2 (s, -CH_2-B-(OR)$_2$).

pLBB(OGly)$_2$, Yield: 3.54 g (68%); ^{1}H NMR (400 MHz, D_2O): δ (ppm) = 3.86-3.36 (m, 28H, O-CH_2-CH_2-O); 3.21 (s, 6H, CO-CH_3); 1.59 (s, 3H, CH_2-C(CH_3)); 1.35 (q, 4H, B-CH_2-CH_2-); 1.28 (sx, 4H, B-CH_2-CH_2-); 0.21 (c, 4H, (CH_2)$_2$-CH_3); 0.74 (t, 12H, B-CH_2).

pLBB(OGlyO6FiP), Yield: 2.16 g (91%); ^{1}H NMR (400 MHz, D_2O): δ (ppm) = 4.41(sp, 1H, B-O-CH(CF_3)$_2$); 3.80-3.46 (m, 16H, O-CH_2-CH_2-O); 3.21 (s, 3H, CO-CH_3); 1.59 (s, 3H, CH_2-C(CH_3)); 1.35 (q, 4H, B-CH_2-CH_2-); 1.28 (sx, 4H, B-CH_2-CH_2-); 0.21 (c, 4H, (CH_2)$_2$-CH_3); 0.74 (t, 12H, B-CH_2); ^{11}B NMR (400 MHz, D_2O): δ (ppm) = 8.2 (s, -CH_2-B-(OR)$_2$); ^{19}F NMR (400 MHz, D_2O): δ (ppm) = -78.2 (s, CF_3).

III. *Additional figures*

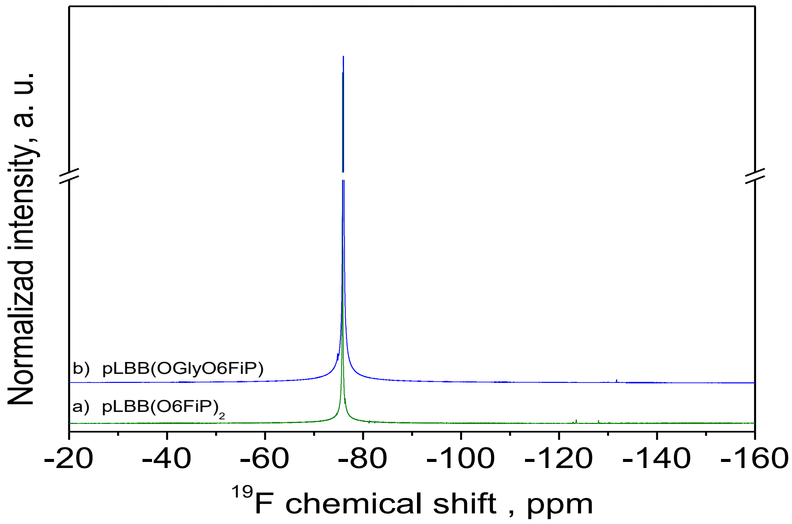

Fig. S1: ^{19}F NMR spectra of SLICPEs: a) pLBB(O6FiP)$_2$, and b) pLBB(OGlyO6FiP).

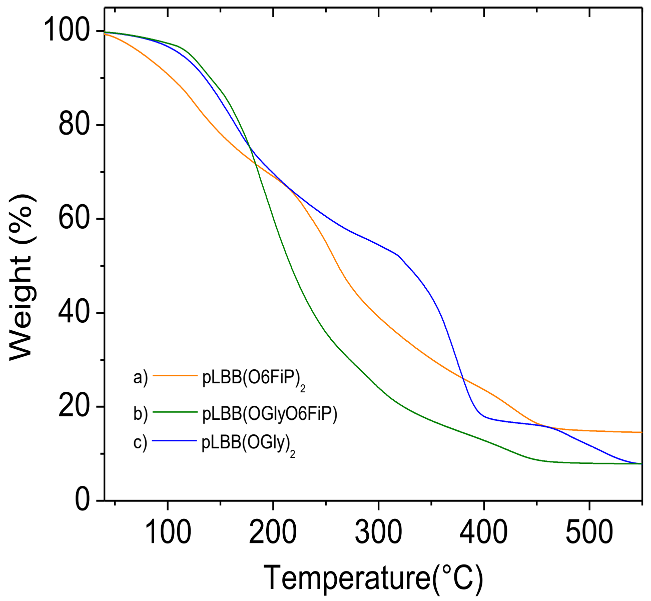

Fig. S2: TGA curves of SLICPEs: a) pLBB(O6FiP)$_2$, b) pLBB(OGlyO6FiP), and c) pLBB(OGly)$_2$

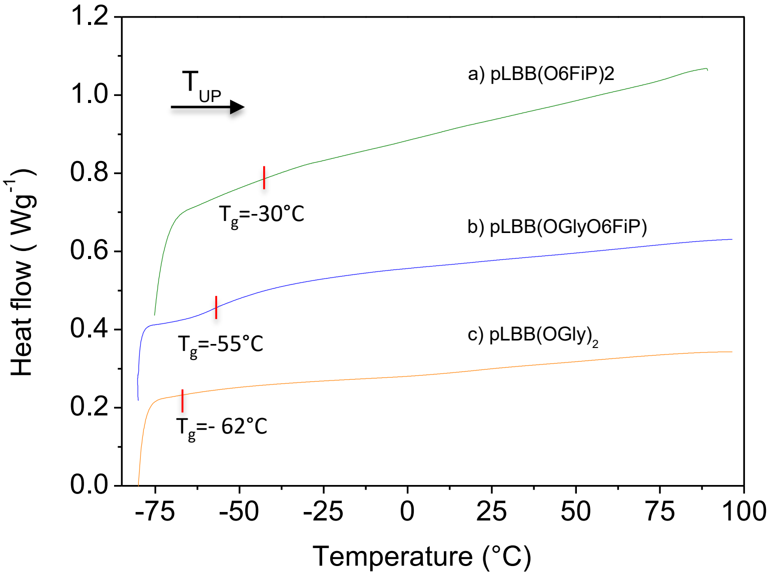

Fig. S3: DSC curves of SLICPEs: a) pLBB(O6FiP)$_2$, b) pLBB(OGly)$_2$, and c) pLBB(OGlyO6FiP).

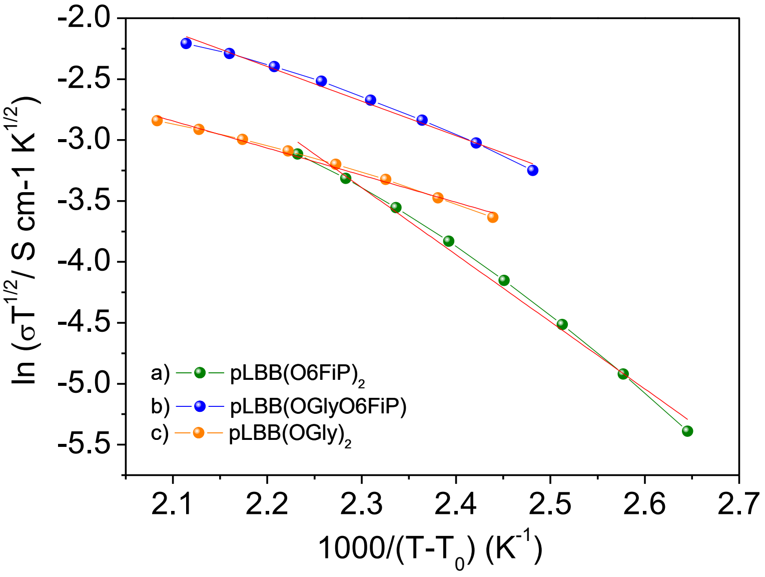

Fig. S4: Temperature dependence of ionic conductivity for SLICPEs Boron-based with several oxy-substituents: a) pLBB(O6FiP)$_2$, b) pLBB(OGlyO6FiP), and c) pLBB(OGly)$_2$. The plots represent the experimental data while the solid lines represent VTF fitting results

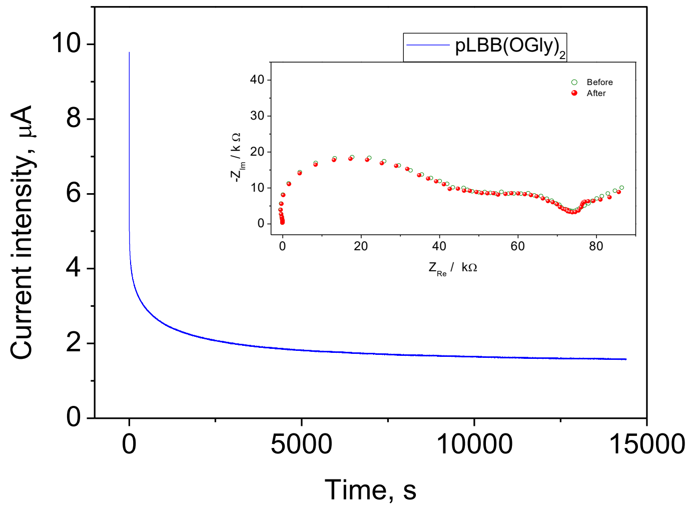

Fig. S5: Lithium transference number (t_{Li^+}) evaluation: typical current transient obtained at polarization of 40 mV for Li^0/ pLBB(OGly)$_2$ /Li^0 cells at 60 °C (inset: Nyquist plot for the same cell before and after polarization).

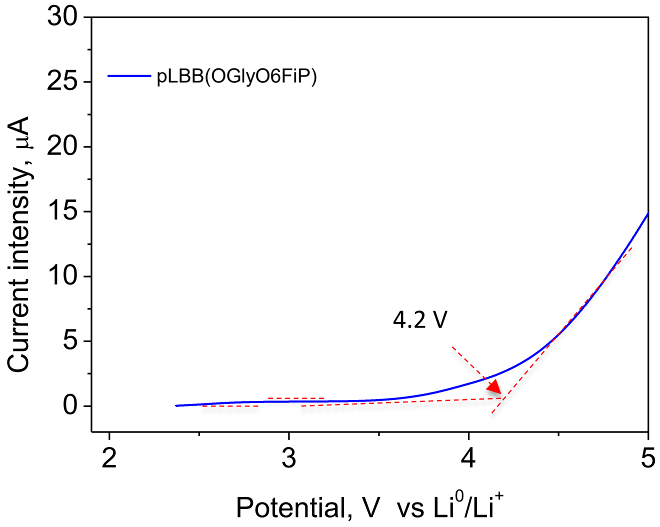

Fig. S6: Linear sweep voltammograms (v = 2 mV s^{-1}) obtained in the Li^0 / pLBB(OGlyO6FiP) / stainless steel cell at 60 °C to evaluate the electrolyte electrochemical stability.

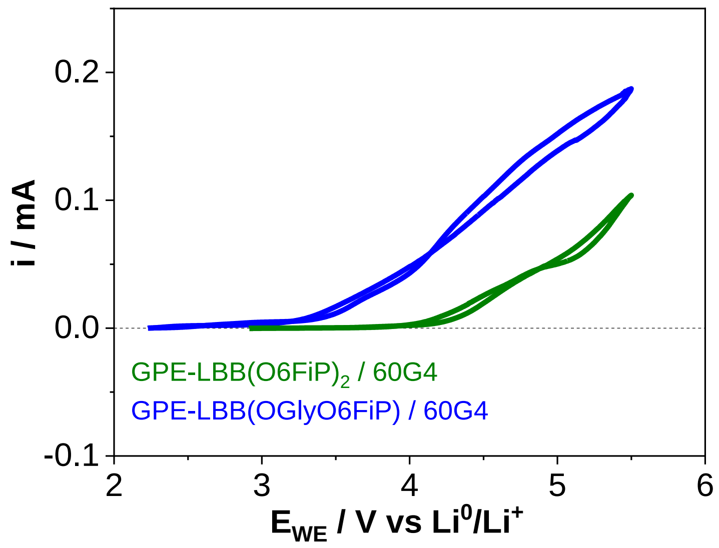

Fig S7. Voltammograms of LBB(OGlyO$_6$FiP)/60G4 and LBB(OFiP)$_2$/60G4 electrolytes at 60 °C and a scan rate of 0.2 mV·s^{-1}. Scans were undertaken in a Li^0/Stainless steel cell from OCV to 5.5 V vs Li^0/Li$^+$. Preliminary results.

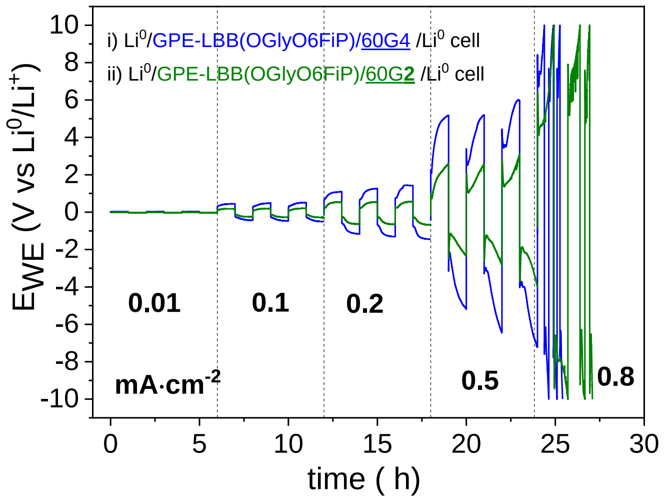

Fig S8. Polarization resistance at different current densities for GPE-BB, using different plasticizers: i) GPE-LBB(OGly6FiP)/60G4 and ii) GPE-LBB(OGly6FiP)/60G2.

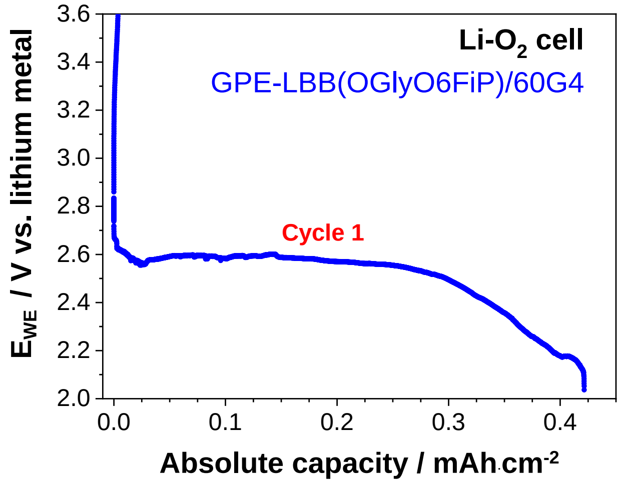

Fig S9. Li-O_2 cells using pLBB(OGlyO6FiP)/60G4 as an electrolyte (preliminary results).

IV. Author Contributions

G. G-G. performed the monomer synthesis experiments and wrote the original draft if not stated elsewhere, S. V. performed the polymers synthesis and electrochemical characterization, M. AT. performed GPE synthesis and electrochemical characterization, S. C. and N. C. supervised the work of S.V., L. C. and A. G. supervised the work of M. AT., and D. M. proposed the topic and supervised the work of G. G-G. and corrected the original draft. All authors discussed the results and reviewed the final manuscript.
